# Keeping Active with Texting after Stroke (KATS): a single-arm feasibility and acceptability study of a behavioural intervention to promote community-based physical activity after stroke rehabilitation

**DOI:** 10.1136/bmjopen-2024-093838

**Published:** 2025-02-08

**Authors:** Jacqui H Morris, Linda A Irvine, Jenna Breckenridge, Albert Farre, Gozde Ozakinci, Keith Jenkinson, Andrew Murphy, Stephan U Dombrowski

**Affiliations:** 1 School of Health Sciences, University of Dundee, Dundee, UK; 2 Faculty of Natural Sciences, Division of Psychology, University of Stirling, Stirling, UK; 3 School of Medicine, University of Dundee, Dundee, UK; 4 Kinesiology, University of New Brunswick Fredericton, Fredericton, New Brunswick, Canada

**Keywords:** REHABILITATION MEDICINE, Behaviour, Physical Fitness, STROKE MEDICINE, Physical Therapy Modalities

## Abstract

**Objectives:**

To test the feasibility and acceptability of a text-message-delivered behavioural intervention to promote and maintain physical activity and recovery after stroke rehabilitation.

**Design:**

A single-arm acceptability and feasibility study.

**Setting:**

Community rehabilitation services in two Health Board areas in Scotland.

**Participants:**

People with stroke who could participate in physical activities and use a mobile phone were recruited during rehabilitation and community rehabilitation.

**Intervention:**

Keeping Active with Texting after Stroke (KATS) is an automated text message-delivered intervention informed by behaviour change theory. It delivers a structured sequence of 103 messages over 14 weeks to support the uptake and maintenance of physical activities following stroke rehabilitation.

**Outcomes:**

Data on recruitment, retention and satisfaction were collected. Semistructured interviews explored intervention acceptability. Preintervention and postintervention measures provided preliminary information on step count, functional independence, mental well-being, self-efficacy and quality of life.

**Results:**

18 men and 13 women were recruited; three withdrew before intervention commencement. All 28 participants who received at least one text message completed the study, indicating 100% retention. Median satisfaction score was 23/25 (range 12–25). All but one participant read and responded to texts, indicating good engagement. Effect sizes (Cohen’s d; per cent change) were demonstrated in step count (0.2; 13%), extended activities of daily living (0.24; 8.3%) and mental well-being (0.35; 7%). Participants perceived KATS as acceptable, valuing messages and motivational prompts, but personalised tailoring was desired by some.

**Conclusions:**

Recruitment, retention and outcome measure completion were feasible, and KATS was perceived as acceptable. Findings suggest some modifications of messages and goal-setting processes are required to accommodate participants with diverse physical activity capabilities before a definitive trial. Promising indicators of effects were detected, although interpretation must be cautious because the study was not powered to determine efficacy, and there was no control group. Based on these findings, KATS will be further optimised before evaluating effectiveness in a randomised controlled trial.

**Trial registration number and protocol availability:**

ISRCTN 13704805 https://www.hra.nhs.uk/planning-and-improving-research/application-summaries/research-summaries/keeping-active-with-texting-after-stroke-kats/

Protocol available https://www.isrctn.com/ISRCTN13704805?q=13704805&filters=&sort=&offset=1&totalResults=1&page=1&pageSize=10

**STRENGTHS AND LIMITATIONS OF THIS STUDY**

- The Keeping Active with Texting after Stroke intervention is theoretically informed and coproduced with people with stroke and rehabilitation professionals.
- Texting as a medium for behaviour change intervention delivery is feasible and acceptable for people with stroke.
- The automated text delivery system aligns with those used in many healthcare systems and can be readily integrated into these systems.
- This mixed methods design provided a comprehensive evaluation of the text message-delivered behavioural intervention.
- This was a small single-arm acceptability and feasibility study with an unblinded assessment; therefore, the findings are preliminary.

## Background

 Stroke is a major cause of adult disability.^1^ 70% of survivors experience physical, communication or cognitive impairments that persist after hospital discharge and limit walking, leisure activities, activities of daily living and quality of life.^2^ These impairments place physical, social, psychological and economic burdens on people with stroke (PWS), families and health services.^1 3^

Maintaining and improving physical recovery and health after stroke involves high-intensity repetition of task-orientated rehabilitation exercises and participation in a range of diverse physical activities for aerobic fitness, strength and balance.^4 5^ Rehabilitation delivered by multidisciplinary teams is the main intervention for physical recovery immediately after stroke; however, it is resource-intensive and time-limited.^6^ After stroke, international guidelines recommend long-term participation in task-orientated exercise and low- to moderate-intensity aerobic activity.^7 8^ The recommendations are underpinned by evidence that engaging in physical activity^9^ and exercise maintains and improves recovery, independence in physical functioning, mobility and balance and cardiovascular health and prevents secondary stroke.^7 10^

Although community-based exercise programmes specifically addressing the needs of PWS can be effective,^11^ participation is often limited by availability, inadequate signposting and referral, cost, transportation and accessibility. Furthermore, evidence from systematic reviews indicates that participation in formal and group exercise programmes does not translate automatically to increases in improved free-living physical activity levels.^9 10^ Consequently, uptake of both postrehabilitation exercise programmes and free-living physical activities is low. Indeed, studies show sedentary time is >78% of waking hours for many PWS, irrespective of physical capabilities,^12^,^15^ indicating the need for interventions to support behaviour change. These should facilitate the uptake and maintenance of free-living physical activity behaviours that are low-cost, accessible, effective and work within diverse community settings.^9 16^ This is especially important, given the barriers to engaging in structured exercise programmes^17^ and reductions in mortality that can be derived for PWS by engaging in free-living physical activities, including walking.^18 19^ Interventions involving theoretically based behavioural change techniques poststroke can increase the uptake and adherence to PA and exercise programmes;^20 21^ however, optimal characteristics and effective delivery modes remain unclear.^9 22^

Text message-delivered behavioural interventions are resource-efficient and effective for promoting the uptake and maintenance of physical activity in clinical populations.^23^ They comprise a series of messages created to promote physical activity. The purpose is to provide structured support to guide people towards behaviour change.^24^ Texting interventions have often been created in line with behaviour change theory, but not always developed with close attention to key theoretical concepts. Text message-delivered interventions are accessible and widely used, even in older populations, and do not require new technologies to receive or use the intervention.^25 26^ However, evidence examining text message-delivered interventions after stroke is limited. Existing studies have focused on multiple behaviours and broad self-management goals^27^ or atheoretical instructions for walking,^28^ neither of which is optimal for long-term physical activity promotion.^29^

To address these limitations, we cocreated the Keeping Active with Texting after Stroke (KATS) intervention with PWS and rehabilitation therapists. KATS is a theory-based behaviour change intervention developed in line with the MRC framework for complex interventions.^30^ Intervention development processes and formative work are reported elsewhere.^31 32^ This single-group pilot study evaluated participants’ perceptions of acceptability and experiences of engaging with KATS and collected data on candidate outcome measures to inform a future randomised controlled trial (RCT).

Specific aims were:

1. To explore the feasibility of recruiting and retaining KATS participants through community rehabilitation services.
2. To examine participant perceptions of acceptability and levels of satisfaction with KATS.
3. To evaluate the feasibility of using selected outcome measures and undertake preliminary exploration of potential effects.
4. To identify required refinements to inform a future RCT.

## Methods

### Design

This was an unblinded non-randomised single-group feasibility and acceptability study.

### Ethical approval

The study was approved by the North of Scotland Research Ethics Committee, study reference number 291 668.

### Patient and public involvement

KATS was coproduced with a patient and public involvement (PPI) group involving PWS who were involved in its conception and commented on all aspects of the study and intervention development. A PPI member (KJ) was part of the research team, contributing to meetings and decision-making and is a coauthor. A wider collaborative working group including rehabilitation therapists, academic researchers and PPI members helped to design the study and met regularly to review the intervention development, progress and dissemination using a structured process developed by a research team member.^33^

### Setting

The study was conducted in community rehabilitation services in two Scottish health board areas.

### Eligibility criteria

Inclusion criteria were stroke diagnosis; over 18 years of age; no medical contraindications to increasing physical activity; able to access and use a mobile phone for text messaging; provide informed consent; able to walk outdoors and/or indoors and willing to participate in the 14-week study at the end of community rehabilitation.

### Participant recruitment

**Figure 1:**
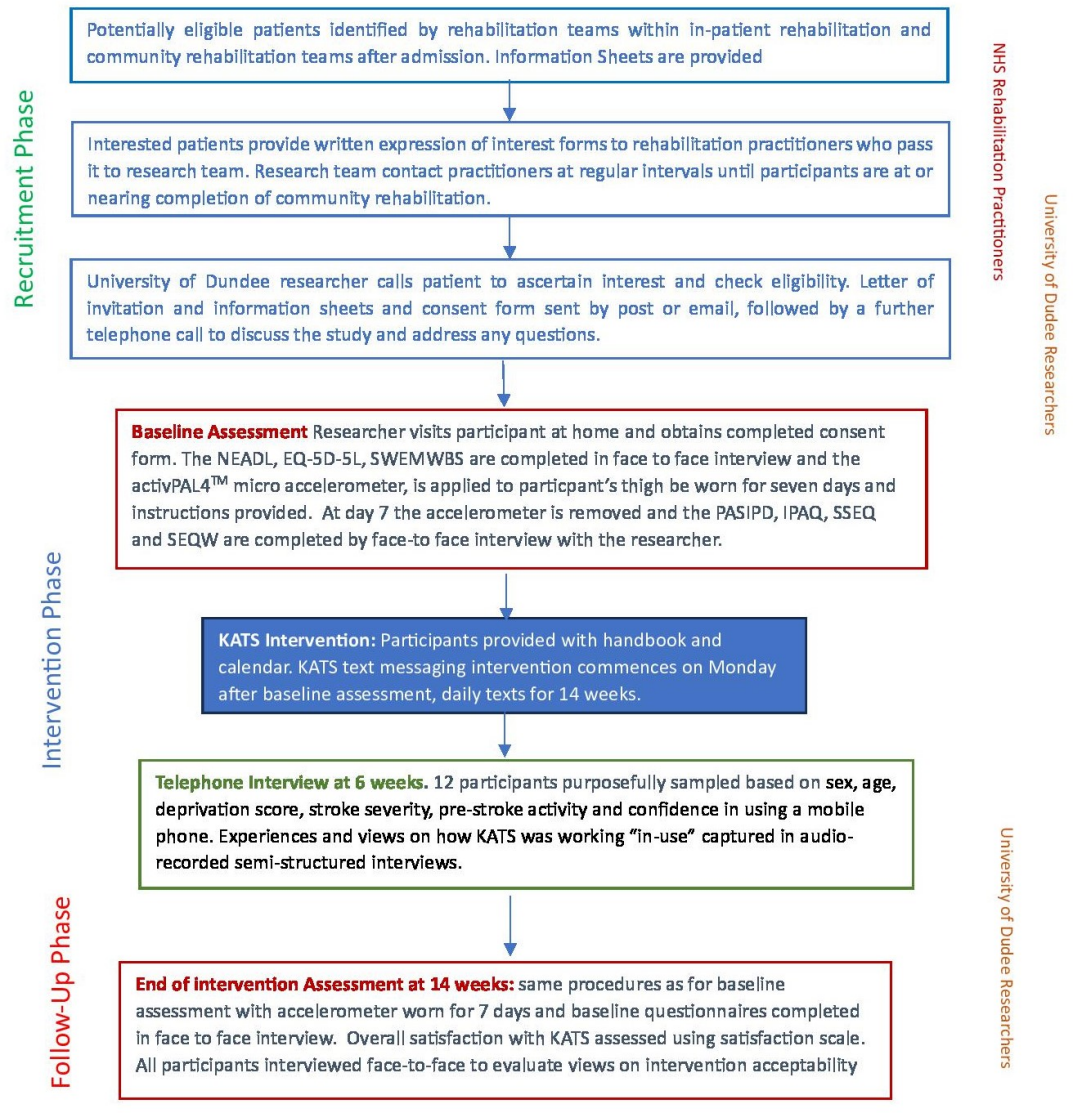
Study flowchart showing the study phases: recruitment, assessment, intervention and follow-up. KATS, Keeping Active with Texting after Stroke; NEADL, Nottingham Extended Activities of Daily Living Scale; PASIPD, Physical Activity Scale for Individuals with Physical Disabilities; SEQW, Self-Efficacy Questionnaire for Walking; SSEQ, Stroke Self-Efficacy Questionnaire; SWEMWBS, Short Warwick-Edinburgh Mental Well-being Scale

Physiotherapists within inpatient and community stroke rehabilitation services identified potential participants from a consecutive cohort of people receiving rehabilitation. People interested in participation provided written expression of interest and permission to be contacted when community rehabilitation ended, or when therapists considered them ready for participation. Researchers then contacted potential participants by telephone to ensure their ongoing interest and study eligibility before providing written informed consent for participation. The participant consent form is provided in the online supplemental appendix 1. Figure 1 provides a flowchart of study procedures.

### Sample size

The study aimed to recruit 30 participants, to provide sufficient quantitative data to evaluate the relevant aspects of feasibility and ensure the qualitative evaluation captured diverse perceptions and experiences.^34^

### Intervention

KATS development processes are reported elsewhere.^31 32^ KATS provides a series of automated messages to enhance motivation, combat feelings of abandonment postrehabilitation and support uptake and maintenance of physical and recovery-specific exercises and activities. Messages were developed to address the needs and concerns of PWS and were personalised to include participants’, researchers’ and rehabilitation therapists’ names. Messages were not tailored to individual needs, but iterative coproduction processes ensured that issues of importance to PWS and rehabilitation professionals were addressed.

**Figure 2:**
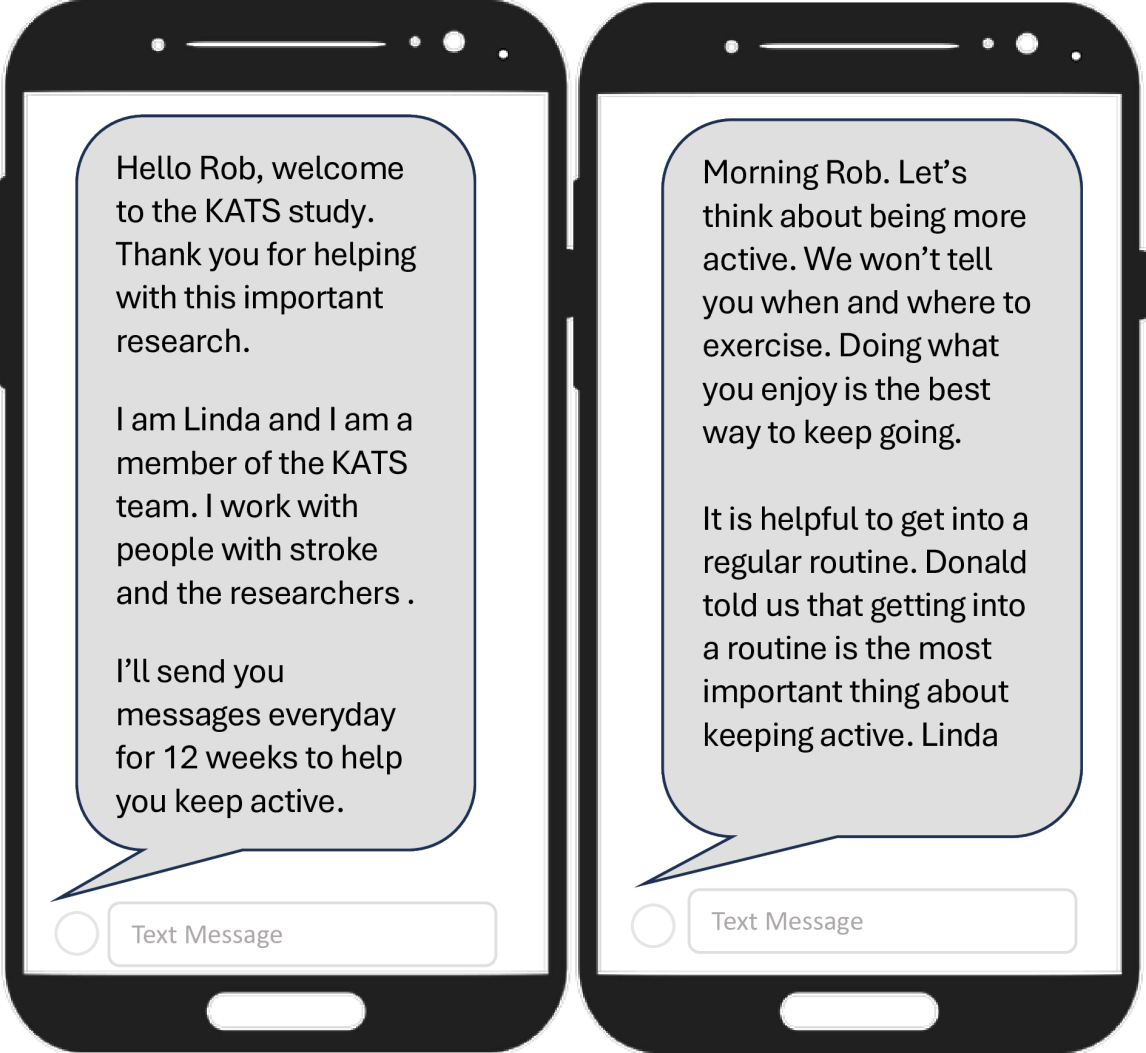
Examples of KATS text messages (OpenClipart- Vectors 2013. iPhone image, https://pixabay.com/vectors/iphone-cell-phone-phone-160307/Pixabay Content License. Accessed 11 December 2023. KATS, Keeping Active with Texting after Stroke.

KATS comprised 103 messages over 14 weeks, delivered by an automated computer system. Messages were organised sequentially according to the Health Action Process Approach (HAPA) constructs^35^ and incorporated relevant behaviour change techniques (BCTs).^36^ Messages were organised into weekly themes, supported by illustrative quotes from PWS, and collated from previous research^37^ and our PPI members. Texts provided real-time support for goal setting, planning and self-monitoring of physical and recovery-specific exercises and activities. Figure 2 provides sample texts, and box 1 describes intervention components according to HAPA. A software tool developed by the Health Informatics Centre at the University of Dundee and used in the team’s previous research^38^,^40^ delivered texts and monitored message delivery. Some messages asked questions to monitor engagement. Participants could reply to texts but were informed the automated system could not deliver personalised replies. The study research fellow received text replies by email in real-time and monitored them daily. In addition to providing data on engagement, this enabled the researcher to identify any problems associated with the study and could intervene if appropriate. Responses to the texts were collected for later analysis. No problems were encountered in delivery and message receipt.

Box 1 **Summary of intervention structure based on HAPA constructs**

**Preliminaries:**

- Introduce Keeping Active with Texting after Stroke (KATS) and its aims and emphasise continuity with rehabilitation and the importance of enjoying activities.

**Motivational phase:**

- Address risk perceptions about exercise and physical activity and the benefits of being active.
- Address outcome expectancies by strengthening the beliefs about positive outcomes of activity after rehabilitation.
- Address action self-efficacy, discuss intentions to be active and reflection on benefits of rehabilitation and goals already achieved.
- Encourage social support through family involvement, providing opportunity to respond to texts and messages from other people with stroke.
- Encourage maintenance by the selection of enjoyable activities congruent with identity and beliefs.

**Volitional phase:**

- Demonstrate goal setting and encourage goals that are congruent with rehabilitation and with desired life goals.
- Provide instructions on action planning including setting cues to action and developing routines.
- Address coping with barriers and introduce self-monitoring strategies for physical activity and the use of diaries.
- Address action self-efficacy by addressing beliefs about capability, reflection on progress and examples from other people with stroke.
- Discuss coping with challenges and developing the capacity to overcome setbacks.
- Reinforce the importance of seeking approval and social support from family and friends.
- Encourage habit formation, establish routines and find ways of building activity into everyday life.
- Encourage maintenance by ensuring that activities are enjoyable and by monitoring and developing strategies to address barriers.

**Maintenance phase**

- Encourage self-monitoring and reflection, address recovery self-efficacy and strategies to recover if they become less active.
- Suggest strategies to cope with adversity, for example, weather, fatigue and the importance of doing activities that they enjoy.
- Encourage social support from family and friends and with supportive texts from people with stroke.
- Encourage habit formation through routines, regular exercise sessions or walking.
- Promote continuing with goal setting and self-monitoring when KATS is finished.
- Encourage reflection on progress and the benefits experienced.
- Draw on physical and psychological resources to encourage self-determination using texts from people with stroke to illustrate and the reflection on achievements since stroke.

Participants also received a paper-based handbook that reinforced key intervention components and provided information and links to online resources offering exercises for PWS. A calendar was provided for self-monitoring activities during intervention delivery for participants to use if they wished. The intervention started the on Monday after the baseline assessment because messages were tailored by the day of the week. On study completion, participants received a gift voucher worth £20.

### Baseline data

Demographic characteristics were collected, including age, sex, details of stroke and recovery, living arrangements, sociodemographic status, current physical activities and use of mobile phones and computers.

### Feasibility and acceptability

Recruitment and retention rates, intervention and outcome measurement completion were examined to evaluate feasibility. To evaluate the perceptions of acceptability, semistructured interviews were conducted by a health services researcher (LI) with 30 years of qualitative research experience within intervention development studies. At 6 weeks, a purposefully selected sample of participants was interviewed, based on sex, age, deprivation score, stroke severity and prestroke activity, to evaluate acceptability ‘in use’. All participants were interviewed at 14 weeks. The Theoretical Framework of Acceptability^41^ for healthcare interventions guided interview schedule development (see online supplemental appendix 2 for Topic Guide), and views were sought on desired changes to KATS. All interviews were recorded and transcribed.

A satisfaction scale^40^ was used at the final assessment to further evaluate the perceptions of KATS (online supplemental appendix 2).

### Outcome measures

Data were collected on outcome measures at baseline and 14 weeks to explore the feasibility of data collection on relevant measures, determine the primary outcome measure for the future RCT, examine the completion rates and detect the signals of potential effectiveness. See study flowchart (figure 1) for the study phases.

Baseline data were collected in two face-to-face visits at participants’ homes to reduce the measurement burden that would have occurred in a single measurement session. Measures included in the first assessment:

- Daily step count over 7 days was assessed using the activPAL4 micro accelerometer, PAL Technologies, Glasgow, UK, as a likely future primary outcome measure. The activPAL^TM^ attaches to the thigh under clothing, on the less-affected leg. The researcher attached the activPAL at baseline, instructing participants in its use. The device was removed on day 8, providing seven complete days of recording.
- The Nottingham Extended Activities of Daily Living Scale (NEADL)^42^ measures disability in activities of daily living. Scores range from 0 to 66; higher scores indicate better performance, with scores of <44 indicating the need for assistance in the activities of daily living^43^
- EQ-5D-5L, EuroQol Research Foundation five dimension assessment of health status,^44^ measures quality of life. The scale has five health dimensions scored between 0 and 5. Higher scores indicate poorer health. The EQ Visual Analogue Scale is a vertical visual analogue scale ranging from 0 mm to 100 mm. 100 represents ‘the best health you can imagine’ and 0 represents ‘the worst health you can imagine’.

After 8 days, the accelerometer was removed, and the following measures were completed:

- The Short Warwick-Edinburgh Mental Well-being Scale (SWEMWBS) measures mental well-being. Scores range from 7 to 35; higher scores reflect better well-being.^45 46^
- The Physical Activity Scale for Individuals with Physical Disabilities (PASIPD), comprising 13 items documenting duration and intensity of participation in leisure, household and occupational activities over the previous 7 days, providing scores in Metabolic Equivalents.^47^
- The Stroke Self-Efficacy Questionnaire (SSEQ) evaluates beliefs in the ability to achieve 13 items on a 10-point scale, where 0=not at all confident; 10=very confident.^48^
- The Self-Efficacy Questionnaire for Walking evaluates the level of walking confidence. Seven items scored between 1 and 5 provide a total score of between 7 and 35; higher scores reflect better self-efficacy.^49^

All measures have established validity and reliability in the context of stroke research (See online supplemental appendix 3 for details of scoring). The sequence of measurement was conducted to minimise the participant burden. Measures were repeated at 14 weeks, and the satisfaction scale was included at that time point.^40^ Detailed information on each measure is provided in the supplementary material.

### Process information

Data on SMS delivery and responses from participants were captured by the automated computer programme, collated and anonymised by the University of Dundee Health Informatics Centre, providing real-time data on study engagement. Detailed field notes were collected at every contact with participants to gather data on recruitment processes, intervention delivery, liaison with participants’ physiotherapists and participant responses to the intervention.

### Quantitative data analysis

Descriptive data were summarised and tabulated, with means and ±SD presented for continuous data. Frequency and percentages were calculated for categorical data. Recruitment and retention rates were determined by the rate of participants providing written informed consent, completing final study assessments and withdrawing, defined as no longer wishing or being able to complete the study. The study was not powered for preintervention and postintervention outcome comparisons; therefore, only between baseline and 14 weeks, mean (SD) and percentage change and effect sizes (Cohen’s d) with 95% CIs on measures were calculated.

### Qualitative data analysis

Qualitative data were managed in NVivo12 and analysed following Braun and Clarke’s approach to Thematic Analysis.^50^ Two researchers (JM and LI) read and familiarised themselves with a selection of transcripts. Transcripts were independently coded using line-by-line open coding, managed in the NVivo 12 software management system.^51^ Initial codes were collated into categories by identification of patterns within the data. These were refined on an ongoing basis through review and discussion by the team, looking for similarities and overlap as data were collected and new transcripts analysed. Finally, interpretive themes on acceptability were sought from the collated codes and categories, guided by the Theoretical Framework of Acceptability (TFA). These were reviewed and agreed on through team discussion. Independent data coding, recording of analytical steps and team discussion ensured transparency, credibility and reliability of interpretation.

### Predefined criteria indicating the feasibility of progression to trial

Table 1 shows our predefined criteria for making decisions about whether to progress to trial, based on recruitment rate scenarios, satisfaction and helpfulness ratings developed by one of the team members.^40^

**Table 1:** Predefined decision criteria

	Recruitment	Retention	Satisfaction rating	Helpfulness rating
Green	>35 participants in 11 months	>85%	>80/100 on satisfaction scale	>4.0/5 on helpfulness scale
Amber	30–35 participants in 11 months	70%–85%	70-85/100 on satisfaction scale	3-4/5 on helpfulness scale
Red	<30 participants in 11 months	<70%	<70/100 on satisfaction scale	<3/5 on helpfulness scale

## Results

### Recruitment and retention

31 participants (target 30) were recruited between mid-November 2021 and mid-September 2022. The prespecified recruitment target for viability of progression to a definitive trial of 30 participants in 11 months was therefore successfully met.

Of the 46 potential participants identified by rehabilitation physiotherapists, 15 (10 male, five female) who were invited to take part declined, indicating a recruitment rate of 66%. Reasons for refusal were not well enough to participate (n=8); progress on recovery from stroke was good so the intervention was not required (n=3); declined without giving a reason (n=3) and one person could not be contacted.

### Participants

Of the 31 recruited participants, 18 were male and 13 were female. Ages ranged from 48 to 84 years (median 71 years) (table 2). 13 participants lived alone. All sociodemographic categories were represented (Scottish Index of Multiple Deprivation scores).^52^ Numbers per quintile, 1 to 5 (1 being the most disadvantaged) were 4,6,2,11 and 8, respectively. Time since stroke ranged from 10 weeks to 89 weeks (median 20 weeks). Three participants withdrew after the first baseline assessment session due to health problems unrelated to the study. 28 participants completed study procedures.

**Table 2:** Baseline: demographic characteristics

	n=31n (%)
Sex	
Male	18 (58)
Female	13 (42)
Living arrangements	
Lives with spouse, partner and/or other family members	18 (58)
Lives alone	13 (42)
Age	
<60 years	6 (19)
60–69 years	9 (29)
70–79 years	13 (42)
>80 years	3 (10)
Range 48–64; mean 67.6; median 71; SD 8.1	
Scottish Index of Multiple Deprivation (SIMD) quintile	
1–2 (most disadvantaged)	10 (32)
3–4	13 (42)
5 (least disadvantaged)	8 (26)
Time from hospital discharge to recruitment	
<10 weeks	10 (32)
11–19 weeks	11 (35)
20–29 weeks	5 (16)
30–39 weeks	2 (6)
>40 weeks	3 (10)
Range 0–156; mean 131.0; median 106.0; SD 73.3	
Time from stroke to recruitment to the KATS study	
<10 weeks	6 (19)
11–19 weeks	9 (29)
20–29 weeks	5 (16)
30–39 weeks	5 (16)
>40 weeks	6 (19)
Range 10–622; mean 180.9; median 137; SD 101.4	

KATSKeeping Active with Texting after Stroke

### Feasibility of outcome measure completion

All questionnaires were completed in full except for the SSEQ, which one participant refused to complete because she considered items were not relevant to her. One accelerometer failed, leading to complete data for 27 participants. Baseline scores are presented in table 3. For this pilot study, we intended to recruit participants with a range of baseline characteristics to investigate how responses varied; therefore, the range of scores on all questionnaires was high (table 3), with daily step counts ranging between 70 and 14 000 steps, indicating a spectrum of physical activity levels.

**Table 3:** Baseline scores on selected outcome measures

	N (%)
**Nottingham Extended Activities of Daily Living Scale (NEADL)**(22 questions, scores 0–66, higher score indicates greater independence, scores <44 indicate needing assistance in Extended Activities of Daily Living)	n=28*
20–39	12 (42)
40–59	14 (50)
>60	5 (18)
Score <44	15 (53)
Range 24–66; mean 44.32; median 47; SD 13.42	
**Physical Activity Scale for Individuals with Physical Disabilities (PASIPD)**(12 questions, scores 0–199.5 MET hours/day, higher score indicates greater intensity of activity)	n=28*
0–5.0	12 (43)
5.1–10	11 (39)
10.1–20	3 (11)
>20	2 (7)
Range 0.17–29.46; mean 7.49; median 6.08; SD 6.98	
**Short Warwick-Edinburgh Mental Well-being Scale (SWEMWBS)**(seven questions, scores 7–35, higher score indicates better mental well-being)	n=31
<20	4 (13)
20–24	6 (19)
25–29	11 (35)
>30	10 (32)
Range 14–33; mean 26; median 26; SD 5.11	
**Stroke Self-efficacy Questionnaire (SSEQ)**(13 questions, scores 0–130, higher score indicates better self-efficacy)	n=28*
<100	9 (32)
100–109	6 (21)
110–119	3 (11)
120–130	10 (36)
Range 53–130; mean 104.71; median 107; SD 21.40	
**Self-efficacy Scale for Walking (SESW)**(seven questions, scores 7–35, higher score indicates better self-efficacy)	n=28*
7–10	4 (14)
11–20	12 (43)
21–30	8 (29)
>30	4 (14)
Range 7–35; mean 18.89; median 16.5; SD 8.37	
**Daily step count, activPal Accelerometer**Higher count indicates greater number of steps	n=27*
<2000	5 (19)
2000–4000	7 (27)
4000–6000	7 (27)
>6000	7 (27)
Range 75–9542; mean 4041.7; median 4164; SD 2014.6	

* Denotes participants completing both baseline assessments.

### Change in outcomes indicating potential effects

Table 4 provides baseline, outcome and change scores, effect sizes and 95% CI for difference in means and percentage changes. The largest effect sizes are for well-being, measured by the SWEMWBS (0.35) and performance of usual activities on the EQ-5D-5L (0.35). There were smaller effect sizes for the NEADL (0.24), EQ-5D-5L visual analogue scale (0.22) and anxiety score (0.21). Percentage change in these outcomes broadly reflects these effect sizes, ranging from 18% on the EQ-5D-5L usual activities scale to 7% on the SWEMWBS. Negative effect sizes and percentage changes were found on the EQ-5D-5L scores on pain (d=−0.17, –8.70%) and mobility (d=−0.04, –1.40%), but these were influenced by large changes in a few individuals.

**Table 4:** Baseline and follow-up scores on selected outcome measures: mean difference (SD); effect size for difference between baseline and follow-up (Cohen’s d); per cent difference between baseline and follow-up

Measure	Baselinemean (SD)(n=28)	Follow-upmean (SD)(n=28)	Mean difference between follow-up and baseline (SD)	Effect size for difference between baseline and follow-up(Cohen’s d); 95% CI	Per cent differencebetween baseline and follow-up
NEADL (min=0, max=22)	44.61 (13.47)	48 (14.57)	3.39 (7.02)	0.24 (–0.76, 0.29)	8.3%
PASIPD MET hour/day (higher score=greater energy expenditure	7.49 (6.98)	7.75 (6.15)	0.26 (5.96)	0.04 (–0.56, 0.48)	3.5%
SWEMWBS (min=7, max=35)	26.79 (4.57)	28.57 (5.59)	1.79 (3.76)	0.35 (–0.61, 0.44)	7.0%
SSEQ* (min=0, max=130)	104.52 (21.78)	107.59 (22.87)	3.07 (14.02)	0.14 (–0.69, 0.42)	4.2%
SEQW (min=7, max=35)	18.89 (8.36)	19.82 (9.77)	0.93 (5.45)	0.10 (–0.61, 0.41)	4.9%
Daily step count (ActivPal)	4041.66 (2543.99)	4575.90 (2937.89)	534.20 (1397.94)	0.20 (–0.70, 0.32)	13.2%
EQ-5D-5L domains (min=1, max=5) lower score indicates better health					
Mobility	2.11 (0.92)	2.14 (0.97)	0.04	−0.04 (–0.54, 0.47)	−1.4%
Self-care	1.75 (1.04)	1.57 (0.88)	0.18	0.19 (–0.33, 0.70)	10.3%
Usual activities	2.29 (1.21)	1.86 (0.97)	0.43	0.35 (–0.13, 0.90)	18.8%
Pain	2.07 (1.05)	2.25 (1.11)	0.18	−0.17 (–0.68, 0.35)	−8.7%
Anxiety	1.75 (1.04)	1.54 (0.96)	0.21	0.21 (–0.31, 0.72)	12.0%
EQ-5D-5L VAS (min=0, max=100)	63.39 (17.75)	68.75 (23.87)	5.36	0.22 (–0.73, 0.29)	15.0%

Note: a higher value at follow-up indicates an improvement in the total score on the following measures: NEADL, Nottingham Extended Activities of Daily Living Scale: higher score indicates better performance; PASIPD, Physical Activity Scale for Individuals with Physical Disabilities: higher score indicates more energy expenditure; SWEMWBS, Short Warwick-Edinburgh Mental Well-being Scale: higher score indicates better wellbeingwell-being; SSEQ, Stroke Self-efficacy Questionnaire: higher score indicates better self-efficacy; SEQW, Self-efficacy Questionnaire for Walking: higher score indicates better self-efficacy; EQ-5D-5L, EuroQuol index of health status. Lower score indicates better health; EQ-5D-5L VAS, EuroroQol 5D-5L Visual Analogue sScale of self-rated health, higher score indicates better health.

* n=27, one participant refused to answer some questions on the SSEQ at follow upfollow-up.

**Figure 3:**
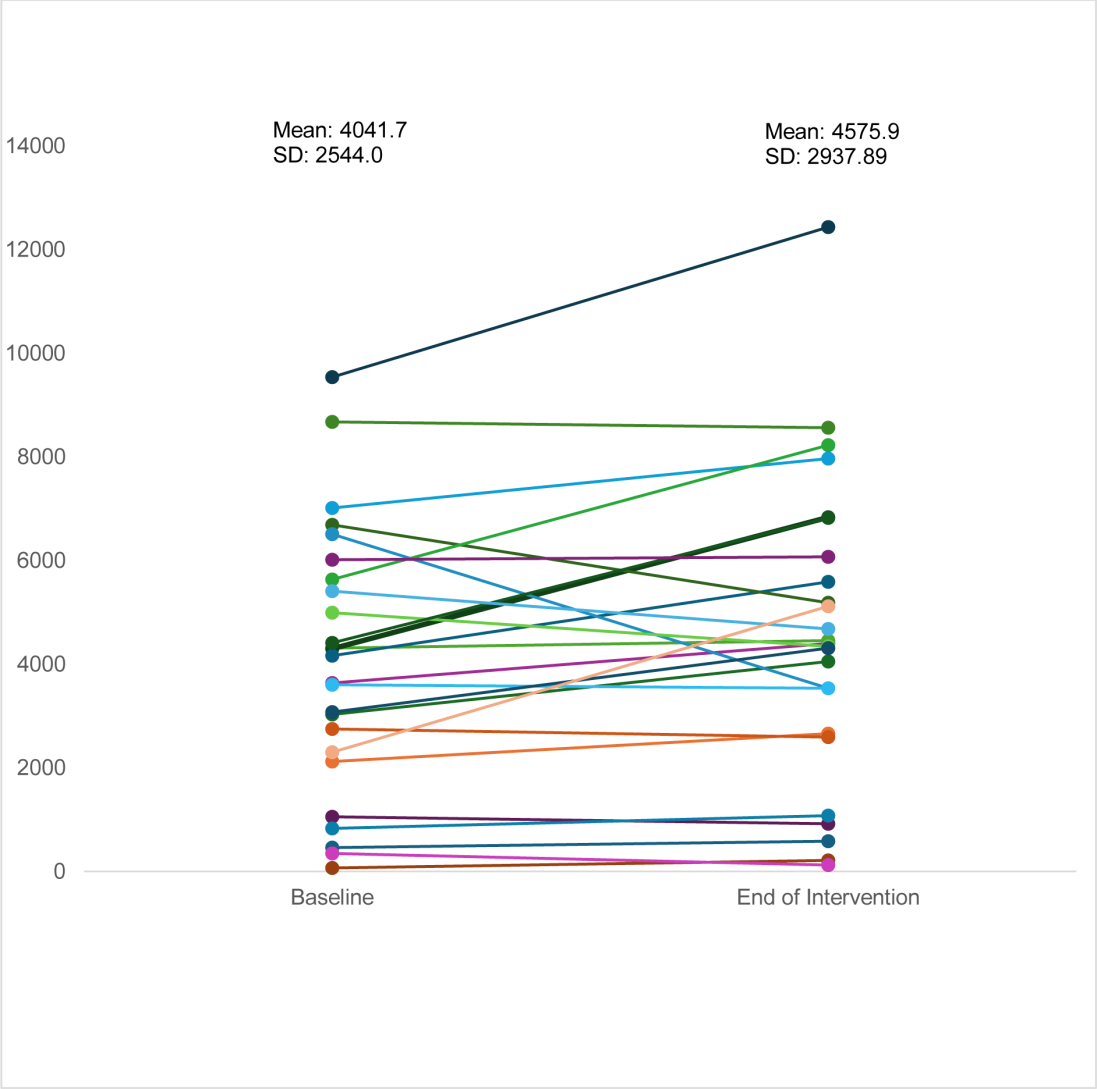
Daily step count at baseline and end of intervention.

**Figure 4:**
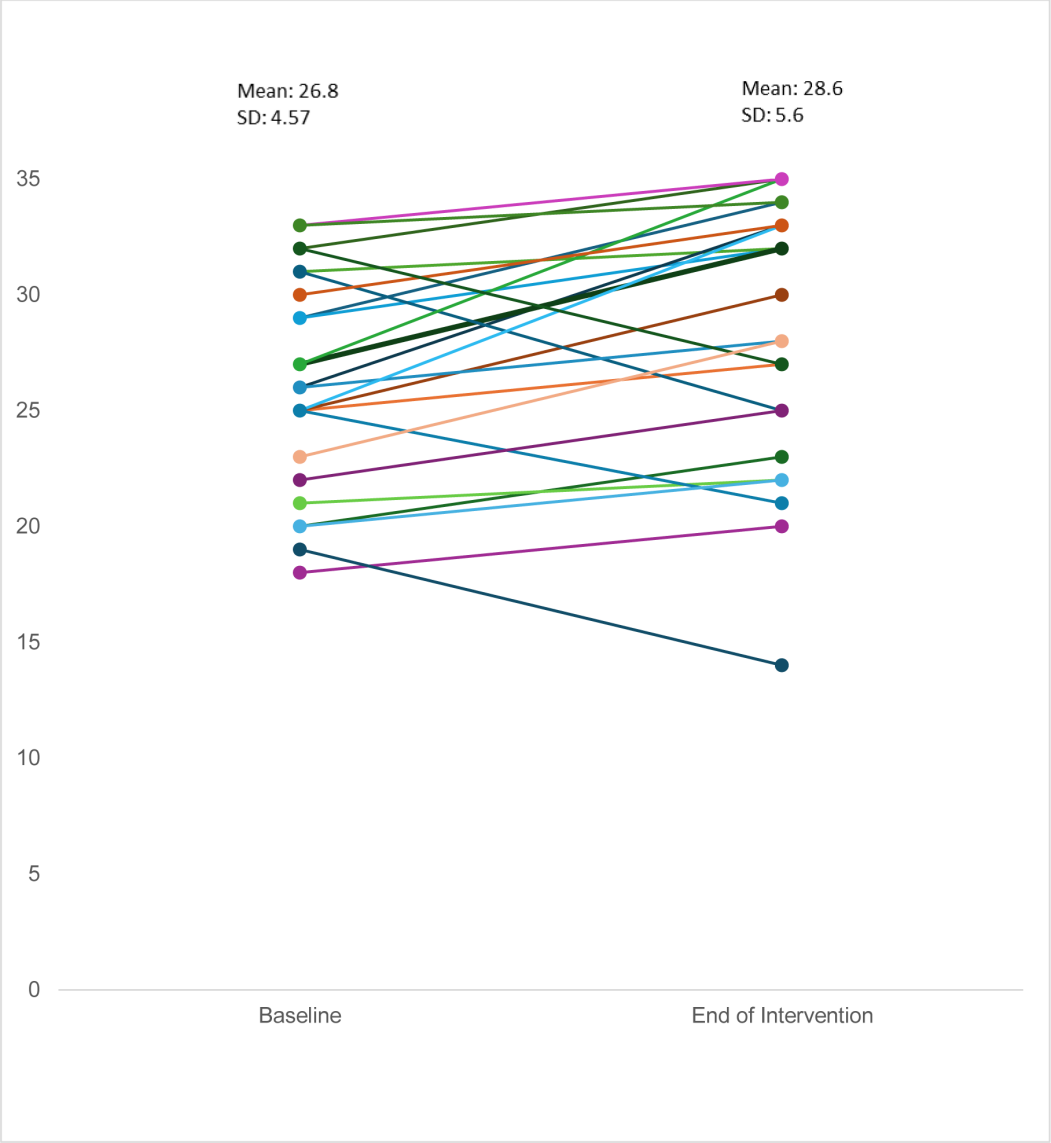
SWEMWBS scores at baseline and end of intervention. SWEMWBS, Short Warwick-Edinburgh Mental Well-being Scale.

Promoting physical activity was a primary aim of this study. There was a mean increase in daily step count of 534.20±2938 (median 147.1), and the effect size for daily step count was 0.2 (−0.70, 0.23). 16 of the 26 participants (62%) improved step counts, 14 (54%) by more than 10%. The last seven participants assessed, of whom all except one had baseline counts >2000 had follow-up assessments in a week of icy weather, which inevitably influenced their outdoor activity and step count. Figure 3 illustrates the change in step count between baseline and end of intervention, and figure 4 the change in SWEMWBS.

### Exploration of acceptability

#### Levels of satisfaction

Mean scores (SD) on the satisfaction scale at follow-up assessment for each domain were Understandable 4.9 (0.4); Useful 4.5 (0.6); Helpful 4.6 (0.6) and Relevant 4.3 (1.0) (scores out of 5). When asked, on a scale of 0–100, how much would you agree with the statement “The KATS programme has helped me achieve activities that are important to me” the average score was 73.3 (19.5). Acceptability and engagement could also be inferred from the high rate of replies to questions sent within the intervention. Participants responded by sending 707 replies across the intervention period, indicating good engagement with KATS. Only one person did not reply at all; responses ranged between 1 and 96, the mean was 25 (±24). Replies reported progress and gave details of new activities. Participants’ text responses to KATS messages were automatically forwarded to the email account of the study researcher. Given this was a remote intervention, messages enabled the team to monitor texts for any adverse events participants reported as resulting from the intervention. Should they occur, the emails would alert the team in case action was required. No adverse events were reported and there were no dropouts or withdrawals because of adverse events related to the intervention. Full analysis of text responses will be reported elsewhere.

Interview data provides information on the qualities of acceptability experienced by participants. Illustrative quotes are provided, and participants are described by sex, age and residual effect of stroke described by the level of independence in complex self-care activities based on baseline NEADL score. Scores of 44 or higher indicate no need for assistance so participants are described as needing or not needing assistance.^43^

Themes derived from data at interim and final interviews show that KATS was valued by most participants for its provision of daily support, but they experienced and used it in different ways that seemed to relate to how the stroke had affected them.

#### Continuity and connection

Messages provided continuity and connection to rehabilitation after it had ended, diminishing the feelings of abandonment. Feelings of isolation and worry of being forgotten in stroke recovery seemed to be alleviated. This sense of connectedness and engagement was reinforced by the daily delivery of texts at different times of day, resembling delivery patterns from a real person.

I think it was good to feel like you were getting a message that there was somebody out there, a connection, that was aware that you had had a stroke, to help you take part and keep going. (Male participant, age early 60s, needing assistance)

#### Using the intervention: planned and prompted

KATS seemed to support the development of self-generated goals and plans, fostering routines and regular activity. Many participants seemed to engage with intervention components as intended, monitoring step counts and reflecting on recovery and activity goals.

I’ve kept a note of everything and when I fill up the diary I’ve been keeping just the steps. If I look up just the numbers at the end of the week, it lets you see how much you’ve been doing. (Male participant, age early 70s, not needing assistance)

However, the activity was not always planned but occurred in response to texts as reminders to do some activity. Messages facilitated spontaneous rather than planned behaviour, generating motivation for activity as real-time nudges rather than planning tools.

Occasionally you did get days when you thought, oh, can I be bothered? And then I’d get a text, and you know, it didn’t matter what it said, “Ah right,” I’ve got all the gear, I should be going out. (Male participant, age mid-70s, not needing assistance)

#### Discerning personal relevance

Quotes from other PWS provided insight into experiences like their own. This was reassuring and provided ideas for new activities. Most understood that KATS was automated and not personally tailored, and they were able to discern suggestions made by other people with stroke from which they could learn most.

Well one of the things is I wasn’t the only person who had a problem. And some people obviously had a lot more problems than me - not that’s comforting to know, but you realise that you’re not alone. (Male participant, age early 60s, not needing assistance)

However, receiving texts that were not relevant to their circumstances could be demotivating for participants who were particularly disabled, because suggested activities could be difficult for them.

Yes, it was all about walking and I thought, well, that’s not for me. I just felt as if I didn’t have anything to contribute to that one. (Female participant, age late 60s, needing assistance)

#### Motivation and modelling

KATS provided ideas for activity, facilitating engagement and determination to do more. Quotes modelled problem-solving and coping strategies and were seen as relevant because they aligned with participants’ experiences and desired improvements. However, a couple of participants with more disability displayed little intention of changing their behaviour despite their willingness to participate in the study. These participants showed ambivalence towards KATS and did not relate to the quotes.

When I’m ready to do it, I’ll do it, I’m not pushing myself too far, I’m no being negative aboot the thing, but I want to get on, but I want to get on in my own time. I dinnae want anybody else’s worries, I’ve got enough of my own. (Male participant, age late 60s, needing assistance)

#### Accessibility and effort

Engagement with KATS was facilitated by the ease of accessibility and availability of text messages, which contrasted with telephone calls. Text messages could be read and re-read at participants’ convenience, giving time to reflect on messages and actions they could take.

Yes, it was so helpful, I do read the texts again, I read them over. I’ve still got them on my phone. I can remember because I cannae keep an eye on them in my head. (Male participant, age early 70s, not needing assistance)

Reading messages was straightforward, but for some, responding could be effortful. Participants with word-finding difficulties or problems with dexterity expressed fear of embarrassment of making spelling mistakes or other errors in sending text messages, creating a reluctance to respond.

If I was replying to something it would probably be full of mistakes because I couldnae see the letters properly, so, I didnae really do a lot of replies. (Female participant, age early 70s, needing assistance)

Overall, the qualitative accounts of acceptability aligned with data on satisfaction, indicating that most found KATS acceptable and useful; however, for some participants, using KATs was more difficult and its relevance to their abilities seemed lower.

## Discussion

Physical activity after a stroke is important for survivor’s health, functioning and well-being.^7 8^ This study shows a texting intervention promoting activity after rehabilitation is feasible and acceptable, and participants found KATS informative and enjoyable. Most participants understood the automated delivery system, discerning which messages were relevant to their own contexts. Some initial signals of potential effect were found, with the greatest change in activities of daily living, well-being and daily step count, outcomes which align with intervention aims. The small sample, single-group design and participant heterogeneity mean only tentative conclusions can be drawn, but the magnitude of change reflects that in other texting studies.^23^

“As a texting intervention KATS is original in addressing the concerns of people with stroke through co-design from inception to delivery” KATS is a theoretically informed intervention developed through collaborative cocreation and refinement processes involving PWS and health and third-sector professionals^33^ and drew on clinical guidelines for physical activity and poststroke recovery. In line with other studies of texting in stroke, this study shows that PWS can engage with and respond to texting interventions.^27 53^ The current qualitative data expands on our early development work which looked at the workability of KATS,^31^ to provide further information about how KATS is experienced and accepted soon after rehabilitation ends. Although most participants accepted the automated nature of KATS, some found the generic messages had limited relevance to them. Tailoring KATS content and delivery by exploring real-time behaviours with ecological momentary assessment to enable us to refine and optimise content at a more granular level will be a focus of further study. However, limited resources meant the researcher who collected quantitative data also conducted interviews, potentially causing social desirability bias. Furthermore, recruitment was conducted during the COVID-19 pandemic and may represent an underestimate of rates under normal circumstances.

KATS participants were encouraged through texts to identify and work towards personal physical activity goals, in alignment with other non-stroke texting studies conducted by the team.^40^ However, qualitative data showed personal goal development was challenging for some KATS participants. Explicitly integrating goal-setting processes into the end of rehabilitation before the commencement of KATS, guided by therapists in line with other stroke texting studies,^27^ is a future development for KATS that could enhance perceptions of the personal relevance of generic KATS messages.

Many people have cognitive and communication impairments after stroke.^2^ Participation in KATS required participants to have access to and be able to use a mobile phone. For this initial pilot study, we only included people who had cognitive and communication abilities that allowed them to read and respond to messages and formulate and enact complex goals. Although we designed the KATS messages with a speech and language therapist to ensure the language used was simple and easily read, the nature of KATS means that in its current form, it may be inaccessible to some people with aphasia and cognitive impairments. To address this issue, and ensure equitable access to the intervention, future developments will incorporate a text-to-voice reader, and we will adapt delivery options to include video messages as well as written texts.

Evidence shows interventions specifically focused on PA, not multiple health behaviours, are more likely to increase PA^29^ which, unlike a recent self-management texting intervention,^27^ was the focus of our study. An RCT of texted walking instructions for people with mild stroke and TIA^28^ showed a mean change in step count of 1500 steps and significant changes in walking capacity. The mean increase of 500 steps/day in KATS (13.2% change) and change in performance of the activities of daily living (NEADL 8.3% change) were comparatively modest but probably reflect the comparatively heterogeneous physical capabilities and physical activity levels of KATS participants. Adapting KATS messaging to account for this heterogeneity and incorporating other motivational and feedback tools into KATS, including wearable technology, may enhance KATS’ potential effectiveness. The potential effects of KATS are likely to reduce the need for hospital admissions and ongoing rehabilitation, making KATS a potentially cost-effective intervention. Future studies will incorporate health economics evaluation. Giving it potential to improve the health of people with stroke in low and middle income countries as well as in high income countries such as the UK.

In line with other texting studies,^54 55^ KATS participants felt supported by the intervention, considered someone cared about them, and found the messages motivational. As with those studies, KATS texts provided reminders that encouraged PA even if participants did not fully engage with all included BCTs.^54^ The volume of responses to KATS texts suggests that engagement was high; however, some participants found creating texts challenging, again suggesting accessible texting methods, including screen readers, and auto voice-to-text technology may be appropriate.

For practitioners, KATS is a low-cost intervention that integrates with rehabilitation goals. Several strategies were identified, however, that could enhance intervention outcomes: developing goal-setting processes for personally relevant goals that better prepare participants for KATS participation; refining and optimising messages most likely to facilitate behaviour change and enhance the motivational aspect of KATS through piloting of wearable technology and feedback. Not all participants showed improved physical activity levels. Future studies will refine messages and augment behaviour change strategies specifically for the least active PWS, who may require a different and more intensive approach. Data show a feasibility RCT is warranted, which will provide information about the feasibility and acceptability of trial processes and provide accurate estimates of effect sizes for a full-scale RCT. Finally, careful creation of a comparator intervention is required, given that any comparison that includes any text messaging may influence outcomes in a population that feels abandoned by services and is seeking support.

## Conclusions

KATS is a feasible and acceptable intervention and recruitment, and retention rates were good. KATs was highly valued by most participants and has the potential to improve physical activity, well-being and activities of daily living outcomes for PWS after rehabilitation. A few areas for further refinement were identified, including refining goal-setting processes with rehabilitation therapists, enhancing motivational aspects of KATS through message refinement and use of wearable technology, adaptations for more disabled PWS and defining a comparator intervention.

## supplementary material

10.1136/bmjopen-2024-093838 online supplemental file 1

## Data Availability

Data are available upon reasonable request.
