# Supplementary material for: Keeping Active with Texting after Stroke (KATS): a single-arm feasibility and acceptability study of a behavioural intervention to promote community-based physical activity after stroke rehabilitation
Source: BMJ Open. 2025 Feb 8;15(2):e093838. doi: 10.1136/bmjopen-2024-093838 (PMC11808871; doi:10.1136/bmjopen-2024-093838)
Supplement: online supplemental file 1 [file bmjopen-15-2-s001.docx]

**Supplemental Materials**

**Appendix 1 Participant Consent Form Template**

**INFORMED CONSENT FORM**

**Appendix 2 Topic Guide and Satisfaction Scale**

Thank you once again for taking part in this study and for agreeing to do this interview today. As I explained at the beginning of the study, we want to find out what you think the messages we’ve been sending out, and how you think they may need to be changed, to help people in the future.

In a few minutes I’ll ask some specific questions about the messages, but first I would like to get your overall impressions about taking part in the study and about the messages you have received:

*Pause to allow the participant to answer without a prompt. If no response, ask:*

How did you find the messages that we sent you?

If the participant goes into detail here – move on to page 2, (**Components of the intervention)** to develop the discussion.

***Timing of the intervention***

(Sometimes these questions fit early, but if the discussion moves straight to page 2, ask these later)

- How did the study fit in with the end of your community rehabilitation?
  - *If the participant says it fitted in well*, can you explain how the messages supported you?
  - *If the participant says it wasn’t particularly helpful*, what type of information could help been helpful?

How did the content of the text messages relate to the work you had been doing with your physiotherapist and occupational therapist?

- - *If it was consistent with rehab,* how did it provide continuity from rehab?
  - *If it wasn’t consistent with what was done*, how did the information/advice differ?
- How did you feel about the timing of receiving the messages after your stroke?
  - If it was too soon, what would have been a better time for you?
  - If it would have been better earlier in your recovery, explain why you would have found useful earlier

**Components of the intervention**

If it is okay with you, I am going to name a few things that we tried to do with the messages and would like you to tell me what you thought about them.

Topics we felt were important to cover included:

- Setting goals to be physically active
- Planning how to achieve goals
- Keeping track of what you, either with a diary or monitoring your physical activity
- Overcoming challenges to keeping active (low motivation, low mood, weather)
- Getting support from your family members and friends
- Texts which gave messages from other people who have had stroke
- Information about access to different types of activity
- How to keep going in the long-term (includes routines and habits)
- Doing things that are important to you

*That way, participants can take each component in the direction they want to and we can check:*

*Recall (do they remember it?)*

*Reflection (do they like it?)*

*Engagement (did they use it?)*

*Impact (did it make a difference?)*

*From here we can also check whether the component worked as intended.*

*Use prompts from below if necessary to guide discussion*

***Goal setting and making plans to be physically active***

- You were familiar with setting goals with your physios and occupational therapists after you had the stroke. We included texts to help you with goal setting and making plans when the therapists’ support ended
- How useful did you find them and why?
- In what ways did you respond to the texts about goal setting/making plans?
- Why did you respond that way?
- Would you change anything about the way we asked you to think about setting goals/making plans?
  - What changes would you make and why?

***Keeping a diary or monitoring your physical activity***

- When we sent out your calendar, we explained that we would not ask to get it back at the end of the study and it was up to you whether or not you used it.
  - Can I ask first of all, did you use the calendar or any other method to record your activities?
  - If you did use it, how useful did you find it and can you describe how it helped you?
  - Would you change anything about the calendar we sent out?
    - What could make it more useful and why?

***Overcome challenges to being active*** (low motivation, low mood, weather)

- Some messages were designed help you to overcome some of the challenges and to help boost your motivation
- Have you been aware that your motivation goes up and down a bit since your stroke (that happens to everyone)?
  - If yes, did you find the messages helpful in boosting motivation?
  - What types of messages did you find most helpful e.g. comments from other people with stroke, encouragement to do a little exercise even if you don’t feel like it

***Getting support from your family members and friends***

- We suggested that you should try to get family members and friends involved.
  - Did you discuss the study with your family or friends?
  - Have you shared any of the messages with them?
  - How are family members and friends helping you to be more active?

***Texts which gave messages from other people who have had stroke***

- Some messages included quotes from other people who had stroke.
- Did you feel these messages were helpful?
  - If yes, how did they help you personally?
- What other information from stroke survivors would be useful?

***Information about access to different types of activity***

- We added information about online resources
  - Did you look up any of the websites we suggested?
    - If yes, how easy was it to find the videos of exercises?
    - How often have you used them?
    - What types of exercise have you tried?
  - Have you tried any other online resources?
    - If yes, what have you found to be useful
  - How could we have made it easier to access the websites?

***Keeping going in the long-term***

- Keeping going in the long-term with physical activity can be challenging
  - What would help you to keep active in the long-term?
  - Do you feel that the activities you are doing are becoming part of your routine or becoming a habit?
    - If yes, what new routines and habits have you developed?

***Doing things that are important to you***

**Text messaging techniques**

***Language used and tone of the messages***

- We wanted the tone to be friendly and encouraging.
  - To what extent do you think we achieved that?
  - Was there anything you would change?
    - If so, what would that be and why?

***Interactive texts*** i.e. answering questions and providing data on activities undertaken

- We ask questions in the texts, and ask you to respond, and to give us information
  - How did you feel about being asked to respond to the questions?
    - How easy or difficult has it been?
  - Is there anything we should do differently?

***Frequency of receiving messages***

- - We sent messages each day, sometimes more than one message.
    - What did you think about the message frequency?
    - Should we change it? If so, how?

***We send the messages out at different times of the day***

- - What do you think is the best time of day for you to receive messages?
  - Why?

***What are your thoughts on the messages with trivia and humour?***

- - Does it add anything, or did you feel it was just an unnecessary distraction?
  - What types of messages would like to see instead?

**Burden of the study/intervention**

- How do you feel about the time and effort required to take part in the study?
  - How much of your time would you say has been taken up with the study so far?
  - How has that time been spent?
  - How does that compare to your expectation at the start of the study?

***The study handbook***

- Does the participant remember the handbook?
  - If yes, when did he/she read it? (just at the beginning, throughout?)
  - How was it used?
  - What other information would have been useful?

**Satisfaction scales**

Finally, can I ask you to think about your experience of the study as a whole:

| Overall the KATS programme was… | | | | | | |
| --- | --- | --- | --- | --- | --- | --- |
|  | | **I  totally disagree** | **I somewhat disagree** | **Neither agree or disagree** | **I somewhat agree** | **I totally agree** |
|  | Understandable | £ | £ | £ | £ | £ |
|  | Useful | £ | £ | £ | £ | £ |
|  | Helpful | £ | £ | £ | £ | £ |
|  | Interesting | £ | £ | £ | £ | £ |
|  | Relevant | £ | £ | £ | £ | £ |

| On a scale of 0 to 100, how much would agree with the following statement, where 0 is not at all and 100 is completely:  The KATS programme has helped me to achieve activities that are important to me |
| --- |
| \|  \|  \| \| \|  \|  \| \|  \|  \| \|  \|  \| \|  \|  \| \|  \|  \| \|  \|  \| \|  \|  \| \|  \|  \| \|  \|  \| \|  \| \| --- \| --- \| --- \| --- \| --- \| --- \| --- \| --- \| --- \| --- \| --- \| --- \| --- \| --- \| --- \| --- \| --- \| --- \| --- \| --- \| --- \| --- \| --- \| --- \| --- \| --- \| --- \| --- \| --- \| --- \| --- \| --- \| \| 0 \| \| \| 10 \| \| \| \| 20 \| \| \| 30 \| \| \| 40 \| \| \| 50 \| \| \| 60 \| \| \| 70 \| \| \| 80 \| \| \| 90 \| \| \| 100 \| \| \| \| *Not at all* \| \| \| \|  \| \| \|  \| \| \|  \| \| \|  \| \| \|  \| \| \|  \| \| \|  \| \| \|  \| \| \|  \| \| \| *Completely* \| \| \| |

What changes would you like to see in the programme?

- Any ideas about how it could be done differently?
- Anything you would want to change, that we haven’t discussed already?

**Appendix 3. Detailed information about Outcome measures**

**The Short Warwick-Edinburgh Mental Well-being Scale (SWEMWBS)**

The Short Warwick-Edinburgh Mental Well-being Scale (SWEMWBS) is widely used to evaluate psychological wellbeing and functioning in the general public and in clinical populations.

The SWEMWBS is an ordinal scale comprising 7 positively phrased items. Items cover aspects of mental wellbeing and answers are provided on a five point Likert scale (“None of the above”, “Rarely”, “Some of the time”, “Often” and “All of the time”). Scores ranges from 7 to 35, with a higher score reflecting a higher level of mental wellbeing. The scale has established levels of validity and reliability in the general adult population (1). These match the psychometric properties of the longer Warwick-Edinburgh Mental Well-being Scale that has been used widely in studies of stroke interventions

**Nottingham Extended activities of Daily Living Scale (NEADL)**

The NEADL measures instrumental functions of daily living after stroke. It comprises 22 items divided into four sections mobility, kitchen, domestic and leisure. Each item is given one of four responses (able, able with difficulty, able with help, unable). These are dichotomized into `independent’ and `not independent’ for scoring. The maximum score is 22 and higher scores equate to greater independence. The scale has been shown to have reasonable hierarchical (ordinal) properties in stroke patients and has established reliability and concurrent validity properties and its sensitivity for measuring EADL has been shown (2-4)

**EQ-5D-5L**

The EQ-5D is a widely used generic instrument for assessing health-related [quality of life](https://www.physio-pedia.com/Quality_of_Life). It is a self-completed questionnaire that describes health state and followed by an evaluation of health state. <https://euroqol.org/eq-5d-instruments/eq-5d-5l-about/>

The respondent classifies his or her prevailing state of health by selecting one of five different levels of problem severity within five dimensions: mobility, self-care, usual activities, pain/discomfort and anxiety/depression. Each dimension has 5 levels: no problems, slight problems, moderate problems, severe problems and extreme problems.

This decision results in a 1-digit number that expresses the level selected for that dimension. The digits for the five dimensions can be combined into a 5-digit number that describes the patient’s health state.

The EQ VAS records the patient’s self-rated health on a vertical visual analogue scale, scored on a 0 to 100 mm scale with 100 representing ‘The best health you can imagine’ and 0 representing ‘The worst health you can imagine’. The VAS is used as a quantitative measure of health outcome that reflect the patient’s own judgement.

The validity of the EQ-5D-5L descriptive system as a generic health outcome measure in PWS is established (5)

**Physical Activity Scale for Individuals with Physical Disabilities**

The Physical Activity Scale for Individuals with Physical Disabilities was developed to assess the self-reported physical activity level of individuals with a disability. It captures information about leisure, household, and work-related physical activity over the preceding 7 days and provides information about the frequency (number of days a week) and duration (daily hours) of physical activity participation. The scale was developed specifically for people with disabilities.

The PASIPD consists of 13 items that document the number of days per week and hours per day of participation in leisure activities, household activities, and occupational activities over the previous 7 days. The scoring process is based on intensity values known as the “metabolic equivalent of the task” (MET). One MET is the ratio of the energy expenditure of an activity over the energy cost of the resting metabolic rate, which is approximately equivalent to consuming one kilocalorie per kilogram of body weight per hour (Taylor et al., 1978). The PASIPD total score is obtained by multiplying the average hours per day spent in each activity, by the MET value associated with the intensity of the activity, and then summing these values to obtain the total score for that week. The maximum possible score on the PASIPD is 199.5 MET hr/day (6). The PASIPD has been evaluated in people with different physical disabilities, demonstrating support for its construct validity, reliability and criterion validity (7) (6).

**The Stroke Self-efficacy Questionnaire**

The SSEQ is a 13-item self-report scale measuring self-efficacy judgements in specific domains of functioning post stroke. Individuals rate their belief in their ability to achieve each of the 13 items on a 10-point scale, where 0 = not at all confident to 10 = very confident. The questionnaire is commonly used in stroke research and demonstrates good psychometric properties (8)

**The Self-Efficacy Questionnaire for Walking**

The self-efficacy questionnaire for walking focuses on walking [in patients](https://www.sciencedirect.com/topics/medicine-and-dentistry/inpatient) with mild [ischemic stroke](https://www.sciencedirect.com/topics/medicine-and-dentistry/brain-ischemia) and [transient ischemic attack](https://www.sciencedirect.com/topics/medicine-and-dentistry/transient-ischemic-attack). The questionnaire includes seven items that are scored using a 5-point Likert-type scale (ranging from 1 to 5 points: 1, not at all confident; 2, not very confident, 3, neutral; 4, confident; 5, very confident). Item scores are totalled, to indicate a total score between 7 and 35, with 35 indicating the highest strength of perceived self-efficacy. The measures show good internal consistency, reliability and criterion validity against established measures of physical activity. (9)

1. Ng Fat L, Scholes S, Boniface S, Mindell J, Stewart-Brown S. Evaluating and establishing national norms for mental wellbeing using the short Warwick–Edinburgh Mental Well-being Scale (SWEMWBS): findings from the Health Survey for England. Quality of Life Research. 2017;26(5):1129-44.

2. Gladman JRF, Lincoln NB, Adams SA. Use of the Extended ADL Scale with Stroke Patients. Age and Ageing. 1993;22(6):419-24.

3. Chong DK-H. Measurement of Instrumental Activities of Daily Living in Stroke. Stroke. 1995;26(6):1119-22.

4. Sarker S-J, Rudd AG, Douiri A, Wolfe CDA. Comparison of 2 Extended Activities of Daily Living Scales With the Barthel Index and Predictors of Their Outcomes. Stroke. 2012;43(5):1362-9.

5. Golicki D, Niewada M, Buczek J, Karlińska A, Kobayashi A, Janssen MF, et al. Validity of EQ-5D-5L in stroke. Qual Life Res. 2015;24(4):845-50.

6. Washburn RA, Zhu W, McAuley E, Frogley M, Figoni SF. The physical activity scale for individuals with physical disabilities: development and evaluation. Arch Phys Med Rehabil. 2002;83(2):193-200.

7. van der Ploeg HP, Streppel KR, van der Beek AJ, van der Woude LH, Vollenbroek-Hutten M, van Mechelen W. The Physical Activity Scale for Individuals with Physical Disabilities: test-retest reliability and comparison with an accelerometer. J Phys Act Health. 2007;4(1):96-100.

8. Jones F, Partridge C, Reid F. The Stroke Self-Efficacy Questionnaire: measuring individual confidence in functional performance after stroke. Journal of Clinical Nursing. 2008;17(7b):244-52.

9. Kawajiri H, Adachi T, Kono Y, Yamada S. Development of a Self-Efficacy Questionnaire for Walking in Patients with Mild Ischemic Stroke. J Stroke Cerebrovasc Dis. 2019;28(2):317-24.
