# Supplementary material for: Keeping Active with Texting after Stroke (KATS): a single-arm feasibility and acceptability study of a behavioural intervention to promote community-based physical activity after stroke rehabilitation
Source: BMJ Open. 2025 Feb 8;15(2):e093838. doi: 10.1136/bmjopen-2024-093838 (PMC11808871; doi:10.1136/bmjopen-2024-093838)
Supplement: Supplementary file 2 [file bmjopen-2024-093838.reviewer_comments.pdf]

PEER REVIEW HISTORY

BMJ Open publishes all reviews undertaken for accepted manuscripts. Reviewers are asked to complete a checklist review form and are provided with free text boxes to elaborate on their assessment. These free text comments are reproduced below.

ARTICLE DETAILS

Title (Provisional)

Keeping Active with Texting after Stroke (KATS): A single-arm feasibility and acceptability study of a behavioural intervention to promote community-based physical activity after stroke rehabilitation

Authors

Morris, Jacqui H.; Irvine, Linda A; Breckenridge, Jenna; Farre, Albert; Ozakinci, Gozde; Jenkinson, Keith; Murphy, Andrew; Dombrowski, Stephan U

VERSION 1 - REVIEW

**Reviewer**	**1**
**Name**	**Tam, Alan**
**Affiliation**	**University of Toronto, Medicine**
**Date**	**02-Nov-2024**
**COI**	**None**

The authors adequately answer the question they pose.

The authors could balance their discussion with a bit more about the limitation of their intervention (reliance on cognitively intact participants, intact language/not aphasic patients). While the most common impairment is motor difficulties, given the benefit of cardiovascular exercise in overall health and secondary prevention, it would be beneficial if the intervention were able to include such populations well, or if not, address it as a limitation.

**Reviewer**	**2**
**Name**	**Saywell, Nicola**
**Affiliation**	**Auckland University of Technology**
**Date**	**07-Nov-2024**
**COI**	**None**

This is an interesting study with potential for translation in to practice at relatively low-cost if found to be effective. I think your use of PPI is great and clearly helped develop an acceptable intervention.

I have mainly minor comments with a couple of more significant points.

Thank you for the opportunity to review the well-written manuscript of a thoughtfully planned and timely study.

Abstract

Line 15

"physical activity activities", sounds clumsy, would "physical activities" be clearer?

Results: I believe one participant did not respond to the text messages, so perhaps change the wording to "All participants read and all but one responded...."

Background

Line 9

I suggest removing the work "impacts".

Lines 30-33

"evidence from systematic review..." needs references to the SRs.

Box 1

I think this all sounds good except for the word "discuss", which suggests a two-way interaction, which is not what this is. The other verbs all seem to describe what I would expect from an automated intervention. If it is a discussion, an example would be helpful.

PASIDIP

I know inactivity is a big problem after stroke, but the scores for this outcome are so low, I just need reassurance that they are accurate!!

General

You have used ‘programs’ and ‘programmes’, ensure consistency of UK or US spelling throughout.

“data was” should be changed to “data were”.

I think that a programme which encourages an increase in activity in people with stroke will collect adverse event data, so I was surprised not to see any record. I see in the CONSORT check list in response to harms, you answered “none”, which implies you did collect these data. I wonder why they were not reported.

VERSION 1 - AUTHOR RESPONSE

Reviewer: 1
Comments to the Author:
The authors adequately answer the question they pose.
The authors could balance their discussion with a bit more about the limitation of their intervention (reliance on cognitively intact participants, intact language/not aphasic patients). While the most common impairment is motor difficulties, given the benefit of cardiovascular exercise in overall health and secondary prevention, it would be beneficial if the intervention were able to include such populations well, or if not, address it as a limitation.

We agree and have added the following text on page 23:
Many people have cognitive and communication impairments after stroke (2). Participation in KATS required participants to have access to and be able to use a mobile phone. For this initial pilot study, we only included people who had sufficient cognitive and communication abilities to allow them to read and respond to messages and to formulate and enact fairly complex goals. Although we designed the KATS messages with a speech and language therapist to ensure the language used was simple and easily read, the nature of KATS means that in its current form, it may be inaccessible to some people with aphasia and cognitive impairments. To address this issue, and ensure equitable access to the intervention, future developments will incorporate a text to voice reader, and we will adapt delivery options to include video messages as well as written texts.

Reviewer: 2
Comments to authors
This is an interesting study with potential for translation in to practice at relatively low-cost if found to be effective. I think your use of PPI is great and clearly helped develop an acceptable intervention. I have mainly minor comments with a couple of more significant points.
Thank you for the opportunity to review the well-written manuscript of a thoughtfully planned and timely study.

Abstract
Line 15
“physical activity activities”, sounds clumsy, would “physical activities” be

clearer?
Now changed

Results: I believe one participant did not respond to the text messages, so perhaps change the wording to "All participants read and all but one responded…."
Now changed

Background
Line 9
I suggest removing the work "impacts".
Now removed

Lines 30-33
"evidence from systematic review…" needs references to the SRs.
Now provided

Box 1
I think this all sounds good except for the word "discuss", which suggests a two-way interaction, which is not what this is. The other
verbs all seem to describe what I would expect from an automated intervention. If it is a discussion, an example would be helpful.

Thank you, we have changed discuss to address

PASIDIP
I know inactivity is a big problem after stroke, but the scores for this outcome are so low, I just need reassurance that they are accurate!!
I have checked the data and it is all accurate. There were items within the PASIPD that were not relevant to many participants, which reflects the low scores. For example, only four participants reported home repairs and outdoor gardening, three reported strenuous sport, caring and work and only one moderate sport. For this mainly elderly stroke population there seems to be a floor effect with this measure and for another study we would consider a measure that was more sensitive to the activities that they undertake.

General
You have used 'programs' and 'programmes', ensure consistency of UK
or US spelling throughout.
Now all changed to programme

"data was" should be changed to "data were".
Corrected

I think that a programme which encourages an increase in activity in people with stroke will collect adverse event data, so I was surprised not to see any record. I see in the CONSORT check list in response to harms, you answered "none", which implies you did collect these data. I wonder why they were not reported.

Thank you, a very good point. We monitored engagement with KATS and adverse events in this

remote intervention through participants' text responses to the KATS messages and questions that we posed. Responses were sent automatically from the automated texts system by email to the study researcher and we monitored them daily for an adverse events or issues of concern. None were reported and there were no withdrawals or drop-outs as a result of adverse events related to the study. We have added the following text to page 23:

Participants' text responses to KATS messages were automatically forwarded to the email account of the study researcher. Given this was a remote intervention, response messages enabled the team to monitor texts for any adverse events participants reported as resulting from the intervention. Should they occur the emails would alert the team in case action was required. No adverse events were reported and there were no drop-outs or withdrawals because of adverse events related to the intervention.
